# Growth in syphilis-exposed and -unexposed uninfected children from birth to 18 months of age in China: a longitudinal study

**DOI:** 10.1038/s41598-019-40134-3

**Published:** 2019-03-13

**Authors:** Huanyuan Luo, Liqian Qiu, Yanqiao Wu, Xiaohui Zhang

**Affiliations:** 1 Department of Epidemiology and Medical Statistics, West China School of Public Health, Sichuan University, Chengdu, China; 2 Department of Women’s Health, Women’s Hospital school of medicine Zhejiang University, Hangzhou, China; 3 Department of Clinical Sciences, Liverpool School of Tropical Medicine (LSTM), Liverpool, UK

## Abstract

Early infant growth and development has attracted worldwide attention. Although numerous studies have demonstrated that maternal syphilis increases the risk of adverse pregnancy outcomes and congenital syphilis, the subsequent growth pattern and morbidity of syphilis-exposed uninfected infants are less understood. We conducted a longitudinal study to compare the growth pattern and disease distribution of syphilis-exposed and syphilis-unexposed uninfected children, and World Health Organization (WHO) reference standards from birth to 18 months of age. We obtained data from a prospective cohort study in three representative regions of Zhejiang Province in China. A total of 333 syphilis-uninfected children born to women with syphilis were recruited at birth and matched with 333 syphilis-uninfected children born to women without syphilis during pregnancy. Children were followed-up by medical staff every 3 months until 18 months of age. The mixed-effects model was used to compare changes in growth patterns and influencing factors between the two groups. Mean weight, length, and head circumference of children, as well as disease prevalence, were similar between the groups. Multilevel analysis indicated that, after controlling confounders, growth velocities were comparable in both weight and length measures from birth to 18 months old between the two groups; however, low birth weight had a negative impact on weight gain in both groups. There was no significant negative association between syphilis exposure and early growth and health in children, under 18 months in a setting with universal coverage of therapeutic interventions for maternal syphilis. These findings may contribute to improving prevention efforts for mother-to-child transmission of syphilis, such as early screening for syphilis in pregnant women, universal coverage of treatment, and interventions for exposed children. Children with low birth weight should be given priority as this is a risk factor for weight gain.

## Introduction

Early infant growth and development play a crucial role in the future health of children. The determinants of infant growth patterns include preterm birth, low birth weight (LBW), maternal characteristics and nutrition, maternal pregnancy indications, infant nutrition, and diseases^1–5^. Although numerous studies have demonstrated that maternal syphilis increases the risk of adverse pregnancy outcomes and congenital syphilis, the subsequent growth pattern and health of infants born to women with syphilis are less well known^6–8^. Currently, considerable research is focused on the growth pattern and health of infants exposed to HIV or HBV^9–14^, in addition to some follow-up studies on congenital syphilis. However, these studies are mainly restricted to disease incidence^15,16^.

There has been a significant reduction in congenital syphilis, and the elimination of mother-to-child transmission of syphilis has been advocated globally^17,18^. Insight into the long-term growth and health of children exposed to syphilis but uninfected is increasingly necessary with the success of effective interventions. There is a significant effort in China to prevent maternal syphilis^19,20^. A national public project for the prevention of mother-to-child transmission of syphilis in China was initiated in 2010^20^. In 2017, the Chinese government implemented a program to eliminate the transmission of syphilis s from mother-to-child in pilot areas. Zhejiang Province is located in eastern China, and the prevalence of syphilis during pregnancy in this province is 0.3%^7^. We previously reported that women with adequate treatment had reduced risk of preterm birth and perinatal disease compared with inadequately treated mothers^7^. Nevertheless, the effects of syphilis in pregnancy on children’s early growth and health are not fully understood. In this research, we conducted a longitudinal study to compare the growth level, growth velocity, and disease incidence between syphilis-exposed and -unexposed uninfected infants, to provide a reference for estimating the effects of syphilis exposure on early growth and health of children.

## Methods

### Study design and participants

This was a longitudinal study using data from a prospective cohort study conducted in three representative cities of Zhejiang Province, China: Jiaxing, Lishui, and Quzhou. The areas were chosen based mainly on the prevalence of gestational syphilis, economic development level, and location. Women diagnosed with syphilis infection in the first trimester during 2014–2015 were included in the study and were matched 1:1 with uninfected women according to maternal age (±5 years) and expected date of delivery (±1 month). We excluded women that were nonresidents or had previous miscarriages, abortions, stillbirths, or multiple births. Children born to these women were followed up until 18 months old. In this study, gestational syphilis was defined as a positive result from both the Toluidine Red Unheated Serum Test (TRUST)/syphilis rapid plasma reagin (RPR) and Treponema pallidum particle agglutination (TPPA)/pallidum hemagglutination assay (TPHA) tests during pregnancy. The study was conducted in accordance with the Declaration of Helsinki. Ethics approval was obtained for this study from the Women’s Hospital School of Medicine at Zhejiang University (2016-0005). All participants included in the study provided their written informed consent. For the participants under the age of 18 years, informed consent was obtained from a parent and/or legal guardian.

### The project of Prevention of Mother-to-child Transmission of Syphilis

China has initiated a national project of prevention of mother-to-children transmission of syphilis. All pregnant women are required to have syphilis screening during their first antenatal care and at delivery. Positive results in both TRUST/RPR and TPPA/TPHA tests indicated gestational syphilis. Women with high risk for syphilis were provided with more frequent screening. Syphilis-positive pregnant women were recommended to immediately have at least two courses of penicillin treatment. Women allergic to penicillin were provided with ceftriaxone as a replacement. Syphilis-positive pregnant women and their infants were routinely followed up by medical staff in local women and children hospitals. We performed congenital syphilis screening for children born to syphilis-positive women at delivery and every three months after birth until 18 months old. Congenital syphilis in an infant was defined as one of the following situations: a 4-fold higher serum titer than that of their mother’s at delivery, laboratory confirmation of Treponema pallidum in clinical specimens by dark-field microscopy, or reactive treponemal antibody test for IgM. All laboratory tests were performed according to national guideline with routine quality controls for reducing false positive or negative outcomes.

### Data collection

A questionnaire was used for data collection. The data included maternal information (age, education, marriage, employment, gravidity, parity, and family income), and children’s information (birth information, disease records, and growth indicators). Maternal and children’s birth information were surveyed by specially trained medical staff in face-to-face interviews at local maternal and child health hospitals. In the follow-up period, children’s weight, length, head circumference, and disease occurrence were also determined and recorded by pediatricians at local maternal and child health hospitals. To assure consistency of the measurement technique, medical staff was trained in conducting the physical examination, taking anthropometric measurements in triplicate, calibrating weighing scales, completing the questionnaires and collecting data. They were routinely trained in Good Clinical Practice similar to methods described by Gibson^21^, and provided with specialized training for this project.

### Statistical analysis

Data were double-entered into Epidata 2.0 and analyzed using SAS 9.2 (SAS Institute, Cary, NC, USA). Significance was considered as *P* < 0.05. We used *t*-tests to compare mean weight, length, and head circumference at birth and at different time points and velocities, and for continuous sociodemographic variables between syphilis-exposed and -unexposed uninfected children. We also used one sample *t*-tests to compared weight, length, and head circumference for children born to women with syphilis using World Health Organization (WHO) reference standards for different months of age^22^. The Pearson’s *χ*^2^ test was used to examine differences in the distribution of disease and categorical sociodemographic variables. The Wilcoxon rank sum test was used for family income.

We established mixed-effects models, controlling for potential confounders to estimate each child’s growth trajectory at monthly intervals. The mixed-effects model is the extension of typical linear regression analysis, which includes random effects in the structure of the mean rather than only fixed effects, and can effectively handle repeated measurements data^23^. Let *i* indicate each measurement at level 1, let *j* indicate each participant or infant at level 2, and the model is as follows:

$$\begin{array}{rcl}{y}_{ij} & = & {\beta }_{0}+{\beta }_{1}A{\rm{ge}}+{\beta }_{2}A{{\rm{ge}}}^{{\rm{2}}}+{\beta }_{3}Group\\  &  & +\,{\beta }_{4}Group\times A{\rm{ge}}+{\beta }_{5}Group\times A{{\rm{ge}}}^{{\rm{2}}}\\  &  & +\,{\beta }_{6}S{\rm{ex}}+{\beta }_{7}S{\rm{ex}}\times A{\rm{ge}}+{\beta }_{8}S{\rm{ex}}\times A{{\rm{ge}}}^{{\rm{2}}}\\  &  & +\,{\beta }_{9}X+({u}_{{\rm{0j}}}+{u}_{{\rm{1j}}}A{\rm{ge}}+{u}_{{\rm{2j}}}A{{\rm{ge}}}^{{\rm{2}}})+{e}_{0ij}.\end{array}$$

The response variable *y*_*ij*_ was either the weight or length measurement. The following variables were included as independent variables: Age (polynomial age/month), Group (children’s exposure condition; 1 = syphilis-exposed, uninfected, 2 = unexposed, uninfected), Sex (children’s sex; 1 = boy, 2 = girl), (Group × Age) and (Sex × Age) interaction terms, and other covariates *X* (maternal education, family income, and underweight at birth), with the random effects on intercept and Age. The variables *u*_0j_, *u*_1j_, and *u*_2j_ were random effects at participant level (level 2), and *e*_0*ij*_ was the general random error at measurement level (level 1). We attempted to include the interaction terms of sex and age in the mixed-effects models because we expected that growth velocities would differ over time between the different sexes. The interaction terms of group and age were added to the models to examine whether syphilis exposure affected growth of children. To better fit the growth pattern over time, we only used data with more than two measurements when building the models.

## Results

### Baseline characteristics

Table 1 shows the baseline characteristics of women and their children included in the study. There was a total of 666 children, including 333 children born to syphilis-positive women and 333 born to syphilis-negative women. The average maternal age was 28.23 ± 5.45 years for syphilis-infected women and 28.44 ± 5.36 years for those uninfected (t = −0.50, *P* = 0.62). Although a larger proportion of syphilis-positive women exhibited high parity compared with syphilis-negative women, it did not reach significance (*χ*^2^ = 2.60, *P* = 0.27). An increased proportion of women with syphilis had primary-level education (*χ*^2^ = 125.38, *P* < 0.01), increased gravidity (*χ*^2^ = 39.09, *P* < 0.01), unstable marital status (*χ*^2^ = 10.33, *P* < 0.01), and unemployment (*χ*^2^ = 18.56, *P* < 0.01) than women without syphilis. In addition, average family income was higher in women without syphilis than those with syphilis *(t* = *−2*.*92*, *P* < *0*.*01)*. Compared with infants born to syphilis-positive women, infants born to women uninfected with syphilis had a similar sex distribution (*χ*^2^ = 0.92, *P* = 0.34), and mean birth weight (t = 0.21, *P* = 0.83) and length (t = −1.61, *P* = 0.11). There was no significant difference in LBW incidence between childbearing women with and without syphilis (*χ*^2^ = 1.41, *P* = 0.24). Women who were not syphilis-infected when recruited remained uninfected at delivery.

**Table 1:** Comparison of baseline characteristics between syphilis-exposed and -unexposed groups.

Characteristics		Syphilis-exposed		Syphilis-unexposed		Statistics	*P*
		n	%	n	%		
**MATERNAL**	Age^**^ (years)	333	28.23 ± 5.45	333	28.44 ± 5.36	*t* = −0.50	0.62
Education	Primary or less	47	14.4	11	3.4	*χ*^2^ = 125.38	**<0**.**01**
	Secondary	257	78.6	169	52.7		
	College or above	23	7.0	141	43.9		
Gravidity	0	56	17.3	120	37.7	*χ*^2^ = 39.09	**<0**.**01**
	1	97	30.0	93	29.3		
	≥2	171	52.7	105	33.0		
Parity	0	182	57.1	190	62.9	*χ*^2^ = 2.60	0.27
	1	127	39.8	106	35.1		
	≥2	10	3.1	6	2.0		
Marital status	Married	313	96.0	320	99.7	*χ*^2^ = 10.33	**<0**.**01**
	Unmarried	13	4.0	1	0.3		
Employment	Employed	194	59.5	242	75.4	*χ*^2^ = 18.56	**<0**.**01**
	Unemployed	132	40.5	79	24.6		
Family income (CNY)^***^		314	60000	315	80000	*Z* = −2.92	**<0**.**01**
**INFANT**	Male	185	57.5	174	53.7	*χ*^2^ = 0.92	0.34
	Birth Weight (kg)^**^	319	3.35 ± 0.51	324	3.35 ± 0.42	*t* = 0.21	0.83
	Low birth weight (**<**2.5 kg)	8	2.5	4	1.2	*χ*^2^ = 1.41	0.24
	Birth Length (cm)^**^	318	49.74 ± 1.92	324	49.95 ± 0.84	*t* = −1.61	0.11

^*^Missing data (loss to follow-up) were not analyzed. ^**^Parameters are Mean ± SD. ^***^Parameters are medians.

Information on syphilis-positive women is shown in Table 2. Of the women with syphilis, 92.65% had latent syphilis, and 88.59% had a serum titer under 1:4 at delivery. Three hundred and fourteen of 333 syphilis-positive women received treatment (treatment coverage of 94.29%). At delivery and throughout the study period, no syphilis-exposed infants were diagnosed with congenital syphilis.

**Table 2:** Information on syphilis-positive women (n = 333).

Characteristics		Number	Percent
Syphilis stage	Latent syphilis	309	92.65%
	I	17	5.11%
	II	5	1.60%
	III	2	0.64%
	IV	0	0
Syphilis titer at diagnosis	<1:4	237	71.17%
	≥1:4	96	28.83%
Syphilis treatment	Yes	314	94.29%
	No	19	5.71%
Syphilis titer at delivery	<1:4	295	88.59%
	≥1:4	38	11.41%

### Growth parameters and velocities at each time point

**Figure 1:**
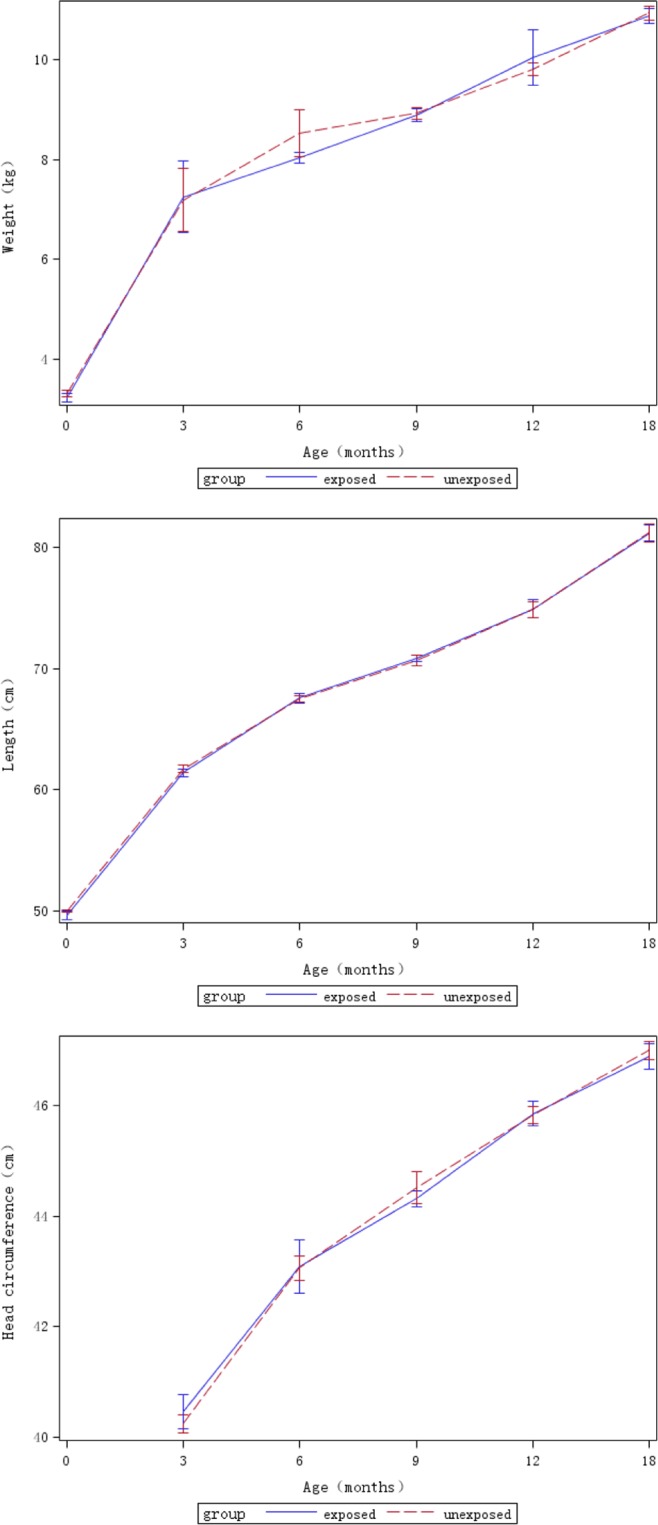
Means (95% confidence interval, CI) of weight, length, and head circumference of children, grouped by syphilis exposure condition, at birth and at 3, 6, 9, 12, and 18 months after birth. Head circumference was not measured at birth.

Mean weight, length, and head circumference (*t*-test, all *P* > 0.05) of children, as well as disease incidence (Pearson’s *χ*^2^ test, all *P* > 0.05) in children were similar between the two groups at 3, 6, 9, 12, and 18 months of age (Table 3). Growth velocities based on weight, length, and head circumference in syphilis-exposed uninfected children were comparable with those in unexposed children at all subsequent time points (*t*-test, all *P* > 0.05), except for mean weight velocity between 6–9 months (*t* = 2.23, *P* = 0.03) (Table 4 and Fig. 1). The greatest changes in weight and length occurred during the first 3 months after birth.

**Table 3:** Comparison of anthropometric parameters, and proportion of diseases in children from the syphilis-exposed and -unexposed groups (mean ± SD, numbers, proportion).

Follow up		Syphilis-exposed	N	Syphilis-unexposed	N	Statistics	*P*
3 months	Weight (kg)	6.52 ± 0.73	281	6.63 ± 0.78	290	*t* = −1.52	0.13
	Length (cm)	61.40 ± 2.75	285	61.71 ± 2.61	292	*t* = −1.19	0.23
	Head circumference (cm)	40.34 ± 1.41	283	40.24 ± 1.37	291	*t* = 0.46	0.64
	Disease n(%)	41(14.6)	281	55(18.9)	291	*χ*^2^ = 1.90	0.17
6 months	Weight (kg)	8.03 ± 0.90	270	8.20 ± 0.99	299	*t* = −1.91	0.06
	Length (cm)	67.55 ± 3.35	270	67.46 ± 2.66	301	*t* = 0.56	0.58
	Head circumference (cm)	42.89 ± 1.86	270	43.05 ± 1.92	299	*t* = −0.77	0.44
	Disease n(%)	44(16.5)	267	52(17.9)	291	*χ*^2^ = 0.19	0.66
9 months	Weight (kg)	8.89 ± 1.13	295	8.94 ± 0.99	310	*t* = −0.63	0.53
	Length (cm)	70.85 ± 2.36	295	70.71 ± 3.69	308	*t* = 0.71	0.48
	Head circumference (cm)	44.32 ± 1.34	295	44.36 ± 1.29	304	*t* = −0.74	0.46
	Disease n(%)	38(13.1)	290	42(14.9)	282	*χ*^2^ = 0.38	0.54
12 months	Weight (kg)	9.76 ± 1.02	266	9.81 ± 1.05	273	*t* = 0.27	0.79
	Length (cm)	74.95 ± 6.10	267	74.89 ± 5.55	274	*t* = 0.35	0.72
	Head circumference (cm)	45.77 ± 1.20	262	45.83 ± 1.25	274	*t* = −0.21	0.83
	Disease n(%)	37(14.5)	255	34(12.8)	265	*χ*^2^ = 0.31	0.58
18 months	Weight (kg)	10.86 ± 1.09	234	10.96 ± 1.13	245	*t* = −0.73	0.46
	Length (cm)	81.18 ± 4.72	233	81.30 ± 5.70	245	*t* = 0.10	0.92
	Head circumference (cm)	46.88 ± 1.78	233	47.02 ± 1.26	245	*t* = −0.64	0.52
	Disease n(%)	27(12.3)	219	25(11.3)	221	*χ*^2^ = 0.11	0.74

^*^Mean ± SD and numbers are calculated using all available data and not only matched data.

**Table 4:** Comparison of growth velocities of anthropometric parameters from syphilis-exposed versus syphilis-unexposed children (mean ± SD, numbers).

Follow up		Syphilis-exposed	N	Syphilis-unexposed	N	*t*	*P*
Birth-3 months	Weight (kg/mo)	1.06 ± 0.28	276	1.09 ± 0.28	281	−1.41	0.16
	Length (cm/mo)	3.89 ± 0.76	273	3.91 ± 0.78	279	−0.14	0.89
3–6 months	Weight (kg/mo)	0.55 ± 0.28	262	0.58 ± 0.32	288	−1.27	0.21
	Length (cm/mo)	2.24 ± 1.16	263	2.18 ± 1.08	289	0.49	0.63
	Head circumference (cm/mo)	0.99 ± 1.29	249	0.95 ± 0.55	271	0.62	0.54
6–9 months	Weight (kg/mo)	0.32 ± 0.29	276	0.28 ± 0.19	289	2.23	**0**.**03**
	Length (cm/mo)	1.24 ± 0.84	283	1.18 ± 0.77	293	1.14	0.25
	Head circumference (cm/mo)	0.53 ± 0.35	275	0.53 ± 0.68	284	<0.001	0.99
9–12 months	Weight (kg/mo)	0.34 ± 0.22	253	0.33 ± 0.20	250	0.84	0.40
	Length (cm/mo)	1.64 ± 0.72	253	1.56 ± 0.67	262	1.46	0.15
	Head circumference (cm/mo)	0.54 ± 0.35	248	0.54 ± 0.34	259	0.75	0.45
12–18 months	Weight (kg/mo)	0.20 ± 0.12	212	0.22 ± 0.14	227	−0.65	0.52
	Length (cm/mo)	1.02 ± 0.31	219	1.10 ± 0.67	233	−1.50	0.13
	Head circumference (cm/mo)	0.22 ± 0.13	211	0.24 ± 0.14	221	−0.80	0.42

### Comparison of WHO standards with growth of children born to women with syphilis

We compared the growth of children born to syphilis-positive women with WHO reference standards, as shown in Table 5. At birth, only girls had greater birth weight and length than the WHO standards (t = 7.33, *P* < 0.001; t = 4.02, *P* = 0.0001, respectively). During follow up, boys in the current study had greater weight in the first year, increased height at 3, 6, and 9 months old, and larger head circumference at 3 and 9 months old compared with WHO standards (*t* test, all *P* < 0.05). Girls in this study had greater weight, length and head circumference at 3, 6 and 12 months, a greater weight at 9 months old, and greater weight and head circumference at 18 months old than WHO standards (*t* test, all *P* < 0.05).

**Table 5:** Comparison of growth between children born to syphilis-positive women and WHO references (Mean ± SD, numbers).

Follow up		Boys	N	WHO-boys	t	*P*	Girls	N	WHO-girls	*t*	*P*
At birth	Weight (kg)	3.33 ± 0.70	18 3	3.35 ± 0.55	−0.46	0.64	3.28 ± 0.51	136	3.23 ± 0.47	7.33	**<0**.**001**
	Length (cm)	49.50 ± 4.09	182	49.88 ± 1.89	−1.24	0.22	49.74 ± 1.00	136	49.15 ± 1.86	4.02	**0**.**0001**
3 M	Weight (kg)	6.73 ± 0.71	148	6.38 ± 0.82	6.01	**<0**.**001**	6.29 ± 0.69	128	5.85 ± 0.75	7.32	**<0**.**001**
	Length (cm)	61.90 ± 2.56	152	61.43 ± 2.04	2.25	**0**.**03**	60.82 ± 2.90	129	59.80 ± 2.11	3.99	**0**.**0001**
	Head circumference (cm)	40.75 ± 1.40	150	40.51 ± 1.18	2.06	**0**.**04**	39.86 ± 1.29	129	39.53 ± 1.24	2.93	**0**.**004**
6 M	Weight (kg)	8.25 ± 0.91	148	7.93 ± 0.87	4.25	**<0**.**001**	7.80 ± 0.82	116	7.30 ± 0.75	6.53	**<0**.**001**
	Length (cm)	68.00 ± 2.29	147	67.62 ± 2.14	2.02	**0**.**04**	66.84 ± 2.98	116	65.73 ± 2.27	4.03	**<0**.**001**
	Head circumference (cm)	43.30 ± 1.53	147	43.33 ± 1.22	−0.24	0.81	42.54 ± 1.12	116	42.20 ± 1.30	3.29	**0**.**001**
9 M	Weight (kg)	9.11 ± 0.93	171	8.90 ± 1.00	2.94	**0**.**004**	8.60 ± 1.31	121	8.23 ± 1.07	3.1	**0**.**002**
	Length (cm)	71.43 ± 2.21	171	71.97 ± 2.24	−3.22	**0**.**002**	70.07 ± 2.35	121	70.14 ± 2.42	−0.32	0.75
	Head circumference (cm)	44.65 ± 1.38	171	45.00 ± 1.26	−3.32	**0**.**001**	43.84 ± 1.08	121	43.83 ± 1.34	0.07	0.95
12 M	Weight (kg)	10.05 ± 0.98	149	9.65 ± 1.15	4.92	**<0**.**001**	9.38 ± 0.96	113	8.95 ± 1.15	4.77	**<0**.**001**
	Length (cm)	76.15 ± 2.48	146	75.75 ± 2.38	1.96	0.05	74.73 ± 2.40	114	74.02 ± 2.58	3.14	**0**.**002**
	Head circumference (cm)	46.14 ± 1.16	146	46.07 ± 1.28	0.72	0.47	45.27 ± 1.07	113	44.90 ± 1.36	3.69	**0**.**0004**
18 M	Weight (kg)	11.04 ± 1.12	127	10.94 ± 1.26	0.97	0.33	10.67 ± 1.04	99	10.23 ± 1.37	4.20	**<0**.**001**
	Length (cm)	82.17 ± 3.20	126	82.26 ± 2.70	−0.31	0.76	80.51 ± 2.82	98	80.71 ± 2.90	−0.69	0.49
	Head circumference (cm)	47.23 ± 1.28	126	47.37 ± 1.33	−1.25	0.21	46.65 ± 1.01	98	46.24 ± 1.38	4.02	**0**.**0001**

### Mixed-effects models for weight and length

To compare the differences in growth pattern between syphilis-exposed and -unexposed uninfected children within 18 months after birth, and to estimate children’s growth trajectories in anthropometric measurements over time, we used the mixed-effects model, controlling for sex, maternal education, family income, and underweight at birth (Table 6). The non-significance of Group terms verified that the mean weight and length of syphilis-exposed and -unexposed uninfected infants at birth were not significantly different (t = 0.58, *P* = 0.56; t = 0.61, *P* = 0.54, respectively). Child Sex terms in the models indicated that the mean birth weight of boys was slightly higher than that of girls (t = −2.14, *P* = 0.03), but no difference was observed in mean length (t = −1.85, *P* = 0.06). The Age terms and Age^2^ terms revealed that the mean weight (t = 30.08, *P* < 0.001; t = −18.96, *P* < 0.001, respectively) and length (t = 21.36, *P* < 0.001; t = −12.03, *P* < 0.001, respectively) of the syphilis-unexposed uninfected infants increased with age, and the growth pattern followed a quadratic curve growth. The (Group × Age) and (Group × Age^2^) interaction terms indicated that, after controlling confounders, growth velocities in the 18 months after birth were not significantly different for both weight (t = 1.60, *P* = 0.11; t = −1.56, *P* = 0.12, respectively) and length (t = −0.76, *P* = 0.44; t = 0.64, *P* = 0.52, respectively) between the two groups, and the growth pattern of syphilis-exposed uninfected infants also showed quadratic curve growth. The (Sex × Age) interaction term demonstrated that boys’ mean weight growth rate was higher than that of girls’ (t = −6.55, *P* < 0.001); consistent with expectations of the interaction between sex and age. The significant random effects of Age terms indicated that age coefficients varied randomly across participants (t = 3.16, *P* < 0.001 for weight, and t = 4.16, *P* < 0.001 for length). Different infants had different velocities because of individual diversity, showing that random effects existed. Therefore, we used the mixed-effects model in this study. Family income and maternal education had no statistical effect on the trend of weight (t = 0.10, *P* = 0.92; t = 0.06, *P* = 0.95, respectively) or length (t = 1.60, *P* = 0.11; t = −1.8, *P* = 0.07, respectively) gain, but LBW had negative impacts on weight gain (t = −5.89, *P* < 0.001).

**Table 6:** Mixed effects models for weight and length in syphilis-exposed and -unexposed children^*^.

Variables	Weight (kg)**					Length (cm)^***^				
	*β*	SE	95% CI	*t*	*P*	*β*	SE	95% CI	*t*	*P*
**Fixed effects**										
Intercept	3.84	0.14	(3.57, 4.11)	28.16	**<**0.001	51.67	0.58	(50.53, 52.81)	89.11	**<**0.001
Group	0.03	0.05	(−0.08, 0.14)	0.58	0.56	0.15	0.25	(−0.34, 0.65)	0.61	0.54
Age (month)	0.97	0.03	(0.90, 1.03)	30.08	**<0**.**001**	3.38	0.16	(3.07, 3.69)	21.36	**<0**.**001**
Age^2^ (month^2^)	−0.03	0.002	(−0.035, −0.028)	−18.96	**<0**.**001**	−0.10	0.008	(−0.11, −0.08)	−12.03	**<0**.**001**
Group × Age	0.02	0.02	(−0.01, 0.05)	1.60	0.11	−0.06	0.08	(−0.21, 0.09)	−0.76	0.44
Group × Age^2^	−0.001	0.0008	(−0.003, 0.0003)	−1.56	0.12	0.002	0.004	(−0.005, 0.01)	0.64	0.52
Sex	−0.11	0.05	(−0.20, −0.009)	−2.14	**0**.**03**	−0.44	0.24	(−0.91, 0.03)	−1.85	0.06
Sex × Age	−0.10	0.02	(−0.13, −0.07)	−6.55	**<0**.**001**	−0.14	0.07	(−0.28, 0.01)	−1.83	0.07
Sex × Age^2^	0.005	0.0008	(0.003, 0.006)	6.08	**<0**.**001**	0.007	0.004	(−0.0007, 0.01)	1.79	0.07
**Maternal education**										
Primary or less	0.006	0.10	(−0.19,0.20)	0.06	0.95	−0.56	0.31	(−1.17, 0.05)	−1.8	0.07
Secondary	0.02	0.06	(−0.10,0.13)	0.28	0.78	−0.13	0.19	(−0.50, 0.24)	−0.70	0.48
College or above	—	—	—	—	—	—	—	—	—	—
Income (CNY)	0.35	3.49	(−6.5,7.19)	0.10	0.92	0.02	0.01	(−0.004, 0.04)	1.60	0.11
Underweight at birth	−1.08	0.18	(−1.44,−0.72)	−5.89	**<0**.**001**	—	—	—	—	—
**Random effects**										
Intercept	**<**0.001	—		—	—	**<**0.001	—		—	—
Age	0.007	0.002		3.16	**<0**.**001**	0.17	0.006		4.16	**<0**.**001**
Age^2^	**<**0.001	**<**0.001		1.43	0.08	**<**0.001	—		—	—
Residual	0.43	0.01		31.46	**<0**.**001**	10.31	0.34		30.74	**<0**.**001**

^*^β = coefficient estimate, SE = standard error. Number of infants = 544 for weight and 539 for length. Number of measurements = 2982 for weight and 2951 for length. Group = 1 for the syphilis-exposed uninfected condition and 2 for the unexposed uninfected condition, sex = 1 for a boy and 2 for a girl.

^**^Adjusted for maternal education (primary or less, secondary, college or above), family income (as continuous variables), and underweight at birth.

^***^Adjusted for maternal education (primary or less, secondary, college or above) and family income (as continuous variables).

## Discussion

The present study compared the differences in long-term growth and health between children exposed to syphilis but uninfected, and a group of children who were not exposed to syphilis. We also compared the growth of uninfected children born to syphilis-positive women with WHO reference standards. In general, the current findings indicated that syphilis exposure did not exert a significant negative influence on growth and health of children from birth to 18 months old. At birth and during the follow-up period, mean weight, length, head circumference, and disease incidence were similar between the two groups of children. Some growth indicators as weight, length and head circumstance in children born to syphilis-positive women were superior to WHO standards^22^, particularly duing 3–12 months after birth. By mixed-effects model analysis, weight and length velocities were comparable between the two groups throughout the study period. Further, LBW exerted a negative influence on weight gain in both groups of children.

In our study, we found no remarkable differences in LBW incidence between childbearing women with and without syphilis. This may be indirectly due to the progress in the policy of Prevention of Mother-to-child Transmission of Syphilis, which promoted syphilis screening and treatment. In the present study, over 90% infected women received syphilis therapy. In addition, a large proportion of women with latent syphilis and lower serum titers also reduced the negative influence on pregnancy outcomes. In this study, 92.65% and 88.59% of syphilis-positive women had latent syphilis and a serum titer under 1:4, respectively. Most studies have reported that women with syphilis, particularly those untreated and with a higher titer (over 1:8), are at higher risk of LBW^6–8,24^.

Currently, the literature on long-term growth of children exposed to syphilis but not infected is limited. There is research similar to ours on infants born to women with HIV or HBV. Some previous studies presented that HIV-exposed uninfected children had lower weight-for-age, length-for-age, and BMI-for-age Z-scores during infancy and through school age than HIV-unexposed children^9,25–27^. A study in the United States (US) found no differences in growth and body composition between uninfected children exposed to HIV and unexposed children under 2 years old; however, uninfected children exposed to HIV had lower birth weights and slightly accelerated growth^27^. The author explained that lifestyle factors may effect growth more than HIV and antiretroviral exposure^27^. However, HIV infection and a higher maternal viral load increases the risk of poor growth^13^. The present results are consistent with those from our previous study of children born to HBV-positive and HBV-negative women, whose growth patterns were comparable during the first 18 months of life^10^. In our previous study, the mother-to-child transmission rate of HBV was 1.0%^10^. A study in Jiangsu showed that children born to HBsAg-positive and HBsAg-negative women had comparable weight and height at ages of 5–7 years, and 3.3% of recruited children were HBsAg-positive and born to HBsAg-positive mothers^14^. Despite the possibility that infants can be infected with HIV, HBV, or syphilis by vertical transmission, these three diseases have different characteristics and intervention measures. Therefore, the growth patterns of HIV or HBV exposed infants only provided references for us.

In our study, both boys and girls born to syphilis-positive women had better growth in some indicators than WHO standards. A previous study also showed that in comparison with the WHO standards, Chinese breastfed infants were heavier and longer on average in the first year of age except from 0 to 3 months old^28^. The data collected by WHO were from widely different settings, and were undertaken during 1997–2003^22^, possibly resulting in some differences. In addition, genetic variations, environmental differences, and social and economic development may also contribute to some different outcomes. Further, Zhejiang is a well-developed economic province in China, promoting optimal health outcomes and growth of children than average levels.

Multiple factors are associated with growth in children, such as maternal nutrition and education level, infant birth weight and disease^29–34^. From the adjusted mixed models, no significant differences in mean weight or length between syphilis-exposed and -unexposed uninfected infants at birth were found, consistent with our *t*-test results. The mixed-effects models also showed that, after controlling for confounders, changes in weight and length were similar in the two groups throughout the first 18 months of age. These findings indicate that maternal syphilis did not have a significant negative influence on infant growth in our study. We also found no significant associations between maternal education and growth of children. In contrast, in central Ethiopia, maternal educational status (adjusted OR: 0.01, 95% CI: 0.001–0.063) showed a negative association with stunted growth in children aged 6–59 months^33^. One study reported that children born to less educated mothers had greater weight and weight-for-length gains from 1–5 years of age than children whose mothers were highly educated, although the underlying mechanisms remained unclear^34^. According to the mixed-effects models, mean birth weight and weight gain of boys were higher than that of girls, but there were no differences in mean birth length and length gain. However, a Tibetan study using a mixed model controlling for weight and length at birth showed no sex difference in weight and length increments in the first year^29^. Nevertheless, the findings of other studies are consistent with our results. A survey performed by the WHO reported that boys were heavier, longer, and had larger head circumferences than girls at birth^35^. In southern India, one birth cohort showed that boys had higher average monthly weight and height gains than girls in the first 1000 days of life^5^. Another study indicated that carotid extra-medial thickness showed a strong association in boys, more so than in girls, that was significantly and positively related to infant weight gain within 18 months of age^36^. The sex difference in physical growth may relate to social factors, such as preferential care and nutrition that boys may receive in developing countries, and physiological factors such as testosterone production^37,38^. Lastly, the current results from mixed effects models found that LBW was negatively associated with the weight gain of infants, supporting previous findings^39–41^. In Northeast Brazil, a cohort study showed that LBW and reduced length at birth resulted in greatly reduced postnatal weight gain and a decreased body mass index and waist circumference of 8-year-old children^39^. In Filipino infants, LBW infants did not catch-up to normal growth parameters by 12 months of age^41^. Therefore, special care is required for newborns with LBW, particularly in the first year after birth.

In this study, no significant difference was seen in disease distribution between the syphilis-exposed and -unexposed uninfected infants. For the syphilis-exposed uninfected infants, clinical presentation of children varied from asymptomatic to acute disease, including severe anemia, hepatosplenomegaly, rhinitis, thrombocytopenia, skeletal damage, and neurosyphilis. To date, comparisons of disease epidemiology between syphilis-exposed and -unexposed uninfected infants has been limited. Several studies have described congenital syphilis symptoms^16,42^. A US study with a large sample size showed a 33.6% morbidity in congenital syphilis cases^16^. In our study, no exposed children were identified as having congenital syphilis.

Our current results suggest that uninfected children born to syphilis-positive women have similar growth patterns and health outcomes as those born to uninfected women from birth to 18 months of age. This may be evidence of the positive achievements of the policy of Prevention of Mother-to-child Transmission of Syphilis in our province. The policy increased awareness of syphilis prevention, and also improved the coverage of syphilis screening and treatment, as well as health care for infants born to syphilis-positive mothers. For example, in the present study, over 90% of syphilis-positive women received treatment, far higher than the earlier provincial level (80.2%) during 2013–2014^7^ and close to the level recommended by the WHO (95%)^43^.

One strength of the current study was the long follow-up period for the syphilis-exposed uninfected children, providing a better understanding of the long-term growth and health of the study population. We also compared the growth of syphilis-exposed uninfected children with reference standards from the WHO. In addition, the mixed-effects model was used to compare the growth patterns of children and influencing factors. This method has been widely used for the estimation of childhood growth^44–47^. Significant random effects in our present results demonstrated the adaptability of the mixed-effects model. Considering the efficiency of the mixed-effects model, it allowed the full usage of information and reduced bias from missing data. The mixed-effects model has the ability to capture heterogeneity in child growth curves and to adequately estimate an individual child’s trajectory^46^. A nonlinear mixed-effects model provides an alternative for analysis of longitudinal data, including power functions or multiplicative relationships to fit growth curves^47^. Covariates and interactions can be added to the model to comprehensively analyze their effects.

Some limitations of this study should also be considered. First, maternal and infant nutrition, and feeding practices were not considered because of insufficient data. Second, the small sample size made further subgroup analyses impossible or lack sufficient power for statistical analysis, such as comparing growth among syphilis-exposed and infected children, exposed but uninfected children, and children born to women treated and untreated. Third, because inadequate goodness-of-fit in a growth model may lead to gross over- or under-estimation of effects, further exploration is warranted of the appropriate growth models to use for the association between syphilis exposure and early childhood growth. Fourth, this study was conducted in areas with high economic development and high coverage of therapeutic interventions for gestational syphilis and frequent antenatal care. And further research into growth and health in school-aged children exposed to syphilis should be undertaken in the future. It is difficult to extrapolate current findings to resource-limited regions lacking intervention. Finally, the possible false positives associated with the misclassification produced by the combination of TRUST plus TPHA might decrease the effect of syphilis exposure on infants’ long-term growth and health.

## Conclusion

There is limited literature on the long-term growth and health of syphilis-exposed uninfected children, particularly in comparison with unexposed uninfected children. Our current results suggest that uninfected children born to syphilis-positive women have similar growth patterns and health outcomes as those born to uninfected women from birth to 18 months of age. This may be evidence of the positive achievements of the policy of Prevention of Mother-to-child Transmission of Syphilis in our province. The policy increased awareness of syphilis prevention, and also improved the coverage of syphilis screening and treatment, as well as health care for infants born to syphilis-positive mothers. Low birth weight should be given priority, as this is a risk factor for the growth of all infants in this study. Our findings should provide a guide or reference to health care policy makers in other countries and regions.

## Data Availability

The datasets are available from the corresponding author on reasonable request.

## References

[1] Simon L (2017). Post-term growth and cognitive development at 5 years of age in preterm children: evidence from a prospective population-based cohort. Plos One.

[2] Black RE (2017). Patterns of growth in early childhood and infectious disease and nutritional determinants. Nestle Nutr Inst Workshop Ser.

[3] Tang L (2017). Maternal lifestyle and nutritional status in relation to pregnancy and infant health outcomes in western China: protocol for a prospective cohort study. Bmj Open.

[4] Hollanders JJ, Sm VDP, Van DP, Rotteveel J, Mjj F (2017). Growth pattern and final height of very preterm vs. very low birth weight infants. Pediatric Research.

[5] Kattula D (2014). The first 1000 days of life: prenatal and postnatal risk factors for morbidity and growth in a birth cohort in southern India. Bmj Open.

[6] Wijesooriya NS (2016). Global burden of maternal and congenital syphilis in 2008 and 2012: a health systems modelling study. Lancet Global Health.

[7] Zhang XH, Xu J, Chen DQ, Guo LF, Qiu LQ (2016). Effectiveness of treatment to improve pregnancy outcomes among women with syphilis in Zhejiang province, China. Sexually Transmitted Infections.

[8] Newman L (2013). Global estimates of syphilis in pregnancy and associated adverse outcomes: analysis of multinational antenatal surveillance data. PLoS Med.

[9] Rosalahallas A, Bartlett JW, Filteau S (2017). Growth of HIV-exposed uninfected, compared with HIV-unexposed, Zambian children: a longitudinal analysis from infancy to school age. Bmc Pediatrics.

[10] Zhang XH (2017). Early physical growth and disease analysis among children born delivered by HBsAg-positive mothers. Zhonghua Yu Fang Yi Xue Za Zhi.

[11] König WJ, Balcha TT, Winqvist N, Björkman P (2017). Growth pattern in Ethiopian infants - the impact of exposure to maternal hiv infection in relation to socio-economic factors. Global Health Action.

[12] Zeng H, Cai H, Wang Y, Shen Y (2015). Growth and development of children prenatally exposed to telbivudine administered for the treatment of chronic hepatitis B in their mothers. International Journal of Infectious Diseases.

[13] Ramokolo V (2014). HIV infection, viral load, low birth weight, and nevirapine are independent influences on growth velocity in HIV-exposed South African infants. Journal of Nutrition.

[14] Chen J, Zhang S, Zhou YH, Xu B, Hu Y (2014). Minimal adverse influence of maternal hepatitis B carrier status on perinatal outcomes and child’s growth. Journal of Maternal-Fetal Medicine.

[15] Vallejo C, Cifuentes Y (2016). Characterization and six-month follow-up on a cohort of newborns with congenital syphilis. Biomedica.

[16] Su JR (2016). Congenital syphilis: Trends in mortality and morbidity in the United States, 1999-2013. American Journal of Obstetrics & Gynecology.

[17] Woodring J (2017). Integrating HIV, hepatitis B and syphilis screening and treatment through the Maternal, Newborn and Child Health platform to reach global elimination targets. Western Pac Surveill Response J.

[18] Taylor M (2017). Elimination of mother-to-child transmission of HIV and syphilis (EMTCT): Process, progress, and program integration. Plos Medicine.

[19] Dou L (2016). Epidemic profile of maternal syphilis in china in 2013. BioMed Research International.

[20] Wang AL (2015). Integrated prevention of mother-to-child transmission for human immunodeficiency virus, syphilis and hepatitis B virus in China. Bull World Health Organ.

[21] Gibson, R. S. *Principles of Nutritional Assessment*. 2nd ed. (Oxford: Oxford University Press, 2005).

[22] World Health Organization, https://www.who.int/childgrowth/standards/en/.

[23] Norleans MX (2001). Statistical methods for clinical trials. Marcel Dekker Inc.

[24] Qin JB (2014). Risk factors for congenital syphilis and adverse pregnancy outcomes in offspring of women with syphilis in Shenzhen, China: a prospective nested case-control study. Sex Transm Dis.

[25] Omoni AO (2017). Child growth according to maternal and child HIV status in Zimbabwe. Pediatric Infectious Disease Journal.

[26] Nicholson L, Chisenga M, Siame J, Kasonka L, Filteau S (2015). Growth and health outcomes at school age in HIV-exposed, uninfected Zambian children: follow-up of two cohorts studied in infancy. BMC Pediatrics.

[27] Neri D (2013). Growth and body composition of uninfected children exposed to HIV: Comparison with a contemporary cohort and US national standards. J Pediatr.

[28] Huang X (2016). Development of a New Growth Standard for Breastfed Chinese Infants: What Is the Difference from the WHO Growth Standards?. Plos One.

[29] Wang W (2016). The growth pattern of Tibetan infants at high altitudes: a cohort study in rural Tibet region. Scientific Reports.

[30] Mallard SR (2014). Dietary diversity at 6 months of age is associated with subsequent growth and mediates the effect of maternal education on infant growth in urban Zambia. Journal of Nutrition.

[31] Ngan M, Durazoarvizu R, Weiss MG, Kramer H (2017). Nutrient-enriched infant formula is associated with higher weight gain for low birth weight infants. J Pediatr Gastroenterol Nutr.

[32] Woon FC (2018). Contribution of early nutrition on the development of malnutrition and allergic diseases in the first year of life: a study protocol for the Mother and Infant Cohort Study (MICOS). BMC Pediatr.

[33] Abeway S, Gebremichael B, Murugan R, Assefa M, Adinew YM (2018). Stunting and its determinants among children aged 6–59 months in northern Ethiopia: a cross-sectional study. J Nutr Metab.

[34] Van DBG, Van EM, Galindo-Garre F, Vrijkotte T, Gemke R (2013). Low maternal education is associated with increased growth velocity in the first year of life and in early childhood: the ABCD study. Eur J Pediatr.

[35] Villar J (2014). International standards for newborn weight, length, and head circumference by gestational age and sex: the Newborn Cross-Sectional Study of the INTERGROWTH-21st Project. Lancet.

[36] Skilton MR (2014). Weight gain in infancy is associated with carotid extra-medial thickness in later childhood. Atherosclerosis.

[37] Vlassoff C (2007). Gender differences in determinants and consequences of health and illness. J Health Popul Nutr.

[38] Smith DW (1976). Shifting linear growth during infancy: illustration of genetic factors in growth from fetal life through infancy. Journal of Pediatrics.

[39] Gonçalves FC, Amorim RJ, Eickmann SH, Lira PI, Lima MC (2014). The influence of low birth weight body proportionality and postnatal weight gain on anthropometric measures of 8-year-old children: a cohort study in Northeast Brazil. Eur J Clin Nutr. 2014 Aug.

[40] Carducci B, Bhutta ZA (2018). Care of the growth-restricted newborn. Best Pract Res Clin Obstet Gynaecol.

[41] Blake RA (2016). LBW and SGA Impact Longitudinal Growth and Nutritional Status of Filipino Infants. PLoS One.

[42] Wozniak, P. S. *et al*. Congenital syphilis in neonates with nonreactive nontreponemal test results. *Journal of Perinatology* **37** (2017). 10.1038/jp.2017.103 28682315

[43] World Health Organization. Global guidance on criteria and processes for validation: elimination of mother-to-child transmission of HIV and syphilis. World Health Organization 2014, http://www.who.int/reproductivehealth/publications/rtis/9789241505888/en/. Accessed 6 Aug (2018).

[44] Howe LD (2016). Linear spline multilevel models for summarising childhood growth trajectories: A guide to their application using examples from five birth cohorts. Statistical Methods in Medical Research.

[45] Yang M, Leung SSF (1994). Weight and length growth of two Chinese infant groups and the seasonal effects on their growth. Annals of Human Biology.

[46] Laird NM, Ware JH (1982). Random-effects models for longitudinal data. Biometrics.

[47] Richard SA, Mccormick BJJ, Miller MA, Caulfield LE, Checkley W (2014). Modeling environmental influences on child growth in the MAL-ED cohort study: opportunities and challenges. Clinical Infectious Diseases An Official Publication of the Infectious Diseases Society of America.

